# A Theoretical Framework and Model for Standardizing Yoga Interventions in Healthcare – MOSI

**DOI:** 10.1177/27536130261446906

**Published:** 2026-04-30

**Authors:** Maria Wahlström, Pär Krutzén, Jenny Krutzén, Eva Hägglund

**Affiliations:** 1 Department of Health Promoting Science, 25548 Sophiahemmet University, Stockholm, Sweden; 2 Swedish Institute Medical Yoga, Oskarström, Sweden; 3 27106 Cardiology Department, Heart and Vascular Center, Karolinska Institute, Stockholm, Sweden

**Keywords:** medical yoga, MOSI, theoretical framework, taxonomy, standardization

## Abstract

This article introduces the MOSI (Medical Yoga Standardized Intervention) theoretical framework, developed to address challenges in the systematic implementation of yoga into healthcare, including intervention heterogeneity and the absence of clinically operational theoretical structures. Informed by long-term clinical application, structured feedback from healthcare professionals, and insights derived from research studies and implementation experience, including patient perspectives – an iterative conceptual analysis and interdisciplinary collaboration guided the development of this framework. It establishes a structured taxonomic derivation and theoretical architecture that operationalizes traditional yoga principles into a clinically applicable structure of a four-layer model comprising four interconnected layers – Self-Care Aspects, Intentional Layer, Practice Layer, and Awareness Layer. The MOSI theoretical framework addresses methodological needs in standardization and implementation, providing a unified language and a structured theoretical foundation to support standardized, reproducible, and clinically applicable yoga intervention design and implementation.

## Introduction

Yoga is a traditional practice with historical roots extending over several millennia and is increasingly studied for its health benefits.^1^ It is recognized internationally as part of humanity’s intangible cultural heritage^2^ and is supported by global initiatives promoting its role in health and well-being.^3^ Despite a growing evidence base across clinical conditions, systematic integration of yoga into conventional healthcare remains challenging. Key barriers include intervention heterogeneity, inconsistent terminology, and limited standardization and clarity in structuring yoga interventions for clinical implementation.^4^ The Medical Yoga Standardized Intervention (MOSI) theoretical framework was developed to address these challenges related to the clinical implementation of yoga interventions.

## Background

The number of published studies on yoga’s health effects has increased significantly,^5^ and multiple systematic reviews and meta-analyses emphasize the efficacy of yoga in improving mental and physical health outcomes, as in depression,^6^ anxiety,^7^ hypertension,^8^ low back pain,^9^ heart failure^10^ and diabetes.^11^ Increased quality of life can be seen in women with breast cancer,^12^ schizophrenia and low back pain.^10^ Yoga can also alleviate fatigue and depression in cancer survivors.^13^ Ye et al identified that yoga improves physical function and disease activity in patients with rheumatoid arthritis.^14^ In terms of cardiovascular diseases, yoga improves outcomes in heart rate and cholesterol^15^ as well as hypertension.^16^ In type 2 diabetes, insulin resistance can be improved after performing yoga.^17^ Yoga also seems to improve lung function and exercise capacity in patients with chronic obstructive pulmonary disease.^18^

Several theoretical and conceptual frameworks have previously been proposed to explain the neurophysiological, psychological, and therapeutic mechanisms underlying yoga-based practices.^19,20^ These contributions have advanced understanding of yoga’s mechanisms and therapeutic effects. However, these frameworks primarily aim to explain mechanisms of action or therapeutic processes and do not define a clinically operational theoretical taxonomy capable of structuring intervention components, standardizing terminology, or supporting reproducible intervention design and implementation. Similarly, CLARIFY 2021, a 21-item checklist developed to standardize the reporting of yoga research, improved transparency by establishing structured reporting guidelines but does not define a theoretical architecture for organizing intervention structures itself.^21^ As a complement to CLARIFY 2021, the MOSI theoretical framework was developed to address this methodological need, to support standardized intervention design, reproducibility and systematic implementation in healthcare contexts.

## Insights

A Swedish form of Medical Yoga, known as MediYoga was established in Sweden in 1997, based on traditional yoga principles.^22^ Programs were adapted to create structured sequences of breathing exercises (pranayama), physical postures (asanas) and meditation (dhyana), targeting various health conditions. Early development focused on refining structured intervention programs and training healthcare professionals to deliver these interventions within clinical settings. This approach enabled integration into routine clinical care, including regular cardiac rehabilitation for patients with myocardial infarction.^22^ Institutional collaboration with academic and healthcare partners enabled systematic clinical evaluation. Clinical studies reported improved quality of life and reduced depression in patients with heart failure^23^ together with improved quality of life and lower blood pressure in patients with paroxysmal atrial fibrillation.^24,25^ Medical yoga has also demonstrated beneficial effects on stress^26,27^ and low-back pain^28^ and has shown potential for delivery through digital platforms.^29^ As implementation expanded, the need for clearer terminology, structured intervention logic and methodological guidance grew stronger as researchers and clinical staff expressed needs for common terminology and clinical guidance.

The most common reported feedback concerned inconsistent terminology, unclear definition of intervention components, and limited guidance for intervention design, dosage, progression, and fidelity. This methodological need directly motivated development of the MOSI theoretical framework.

In addition, discrepancies between traditional non-clinical explanatory models within the yoga field and the conceptual frameworks used in healthcare created challenges in education, interdisciplinary communication, and guideline development.

## Definition of Concepts

Yoga has been defined and interpreted in various ways across ancient and modern texts. One of the most widely recognized traditional sources is the Patanjali Yoga Sutras.^30^ Within this system, yoga is described as comprising eight interconnected limbs: Yama, Niyama, Asana, Pranayama, Pratyahara, Dharana, Dhyana, and Samadhi which together form a comprehensive traditional theoretical system for understanding the multidimensional nature of yoga practice.^1^ This multifaceted system, due to its conceptual complexity, has been interpreted and adapted into numerous yoga traditions across different cultural and clinical contexts. As yoga becomes increasingly integrated into healthcare, clear taxonomy and standardized definitions are essential to support consistent interpretation, communication, and implementation. A theoretical taxonomy provides a structured conceptual architecture that facilitates the operationalization of traditional yoga principles into clinically applicable formats. Foundational definitions of yoga, yoga therapy, and medical yoga are summarized in Table 1 to illustrate the breadth, overlap, and variability in how these terms are defined and applied across traditional and clinical contexts. They do not constitute the taxonomy itself but provide an essential conceptual basis for the MOSI theoretical framework.

**Table 1.** Key Definitions of Yoga, Including Traditional Yoga, Yoga Therapy and Medical Yoga

**Yoga** is a discipline first described 5000 years ago, derived from the Sanskrit term ‘Yuj,’ meaning ‘to yoke’ or ‘to unite’^1^ Traditional yoga, described in the Patanjali Yoga Sutras, encompasses eight elements forming the eight limbs of Yoga, referred to as the Patanjali Yoga System definition (PYSD) in this manuscript.
**Yoga** **therapy** may be defined as, “*the application of yogic principles to achieve physical, physiological, psychological, social, and spiritual well-being*”^1^
**Medical** **yoga** is a collective term with different variations and definitions pointing to the use of yoga for health. A commonly cited definition of medical yoga is “*The use of yoga practices for the prevention and potential treatment of medical conditions*.”^31^ one form of medical yoga, known as MediYoga has been widely used in Swedish healthcare and evaluated in clinical studies.

## Development Process

The MOSI theoretical framework was developed through a structured, implementation-informed conceptual derivation process integrating traditional yoga theory (the eight-limbed system) with clinical requirements for standardized and reproducible interventions. Development was informed by long-term (2016-2025) clinical implementation of structured yoga programs and instructor training initiatives in Swedish healthcare, as well as research collaborations evaluating feasibility, acceptability, and clinical outcomes.

This process involved interdisciplinary collaboration among healthcare professionals (physicians, physiotherapists, nurses), patient organizations, clinical researchers, and experienced yoga therapists. Interdisciplinary working groups representing these collaborators reviewed implementation experiences and identified recurring operational challenges throughout the development process.

Development progressed through iterative development and refinement cycles involving:

1. Clinical observation and identification of implementation challenges
2. Conceptual analysis and theoretical mapping
3. Framework structuring and component definition
4. Iterative refinement through application and conceptual clarification

Through these iterative cycles, the conceptual structure of MOSI gradually evolved into a taxonomy of intervention components, operational terminology, and an integrated theoretical model intended to support clinical usability while preserving the integrity of core yogic concepts.^32^ This translational process involved the ongoing adaptation of traditional yogic concepts, explanatory models, and instructional approaches into clinically relevant definitions, practical guidelines, and patient-appropriate modes of delivery. The development process also informed the broader evolution of MOSI through creation of practical instructor guidelines, training materials, and digital tools designed to support consistent implementation of MOSI-based interventions in clinical settings.

## Theoretical Framework and Model

**Figure 1.**
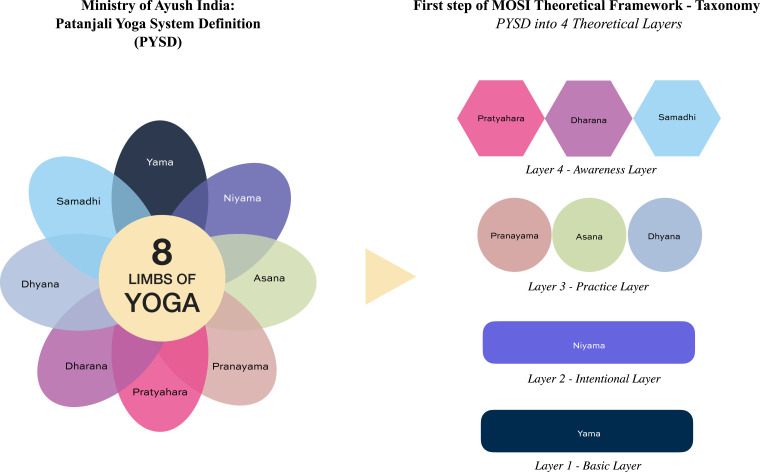
Taxonomy of the PYSD organized into four interconnected theoretical layers

The MOSI theoretical framework organizes principles from the eight limbs of yoga^1^ into a structured conceptual architecture designed to support clinical application. Within the framework, these principles are organized into four interconnected theoretical layers, Basic Layer, Intentional Layer, Practice Layer, and Awareness Layer (Figure 1).

## Interconnected Layers

**Figure 2.**
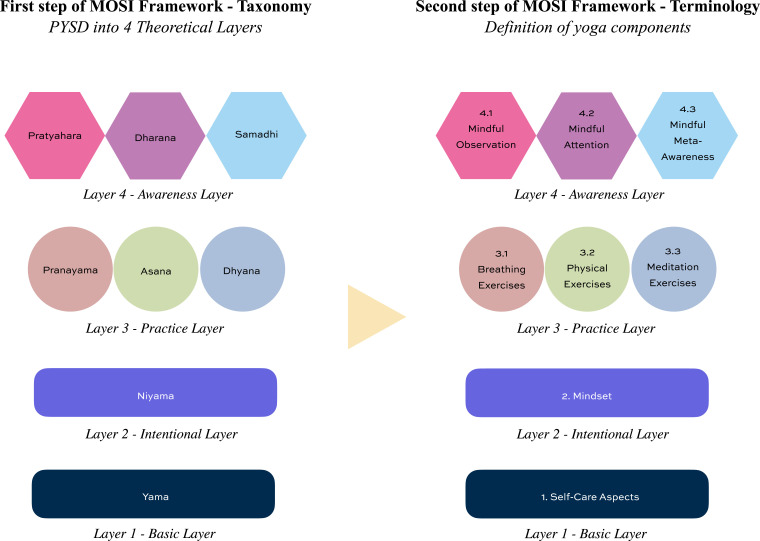
From PYSD taxonomy layers to clinical component definitions and terminology

Definitions for each component and layer of the framework are informed by traditional yoga descriptions and mapped into clinically operational terminology, as illustrated in Figure 2. Each component (1-8) is subsequently described in detail in the following sections.

### Basic Layer-Self-Care Aspects (Niyamas)

In yoga, the Niyamas are a foundational element providing recommendations to stay in balance to avoid disturbances in the process.^1^ The basic layer focuses on Self-Care Aspects that emphasize reflection on behavioral and lifestyle factors that could influence practice and outcomes. It promotes positive self-care behaviors while reducing barriers that affect regulation, quality and results.^33^ The components are maintaining health, positive reappraisal, adherence to routine, monitoring by reflection and self-efficacy. These are standards in the MOSI framework but can be adjusted, promoted and specified in various settings.

### Intentional Layer - Mindset (Yamas)

In yoga, the Yamas represent foundational principles, encompassing non-violence, truthfulness, non-stealing, moderation of the senses and non-greed.^34^ The intentional layer serves both to direct mental aims, such as compassion and acceptance, but also to be integrated into exercises that cultivate these mental qualities over time. This layer forms the psychological foundation, underpinning all exercises to enhance psychological resilience and support mental well-being. The mindset is cultivated by emphasizing compassion, truth, non-judgment, acceptance, and an attitude of gratitude. These qualities serve as essential internal components, to present awareness and foster a stable, reflective mental state. By cultivating these attributes, practitioners may be empowered to cope more effectively with challenges, promoting a salutogenic, safe, person-centered approach that reinforces growth and healing.^35-38^

### Practice Layer - Exercises

The third interconnected layer encompasses three core exercise categories, implemented progressively and stepwise. Informed by clinical experience across diverse patient populations, this approach emphasizes feasibility and accessibility, including exercises for individuals with no prior mind–body intervention experience to support gradual development.

#### Breathing Exercises (Pranayama)

In yoga, “*Pranayama can be referred as the breathing techniques that steady the body and mind*.”^1^ In this framework, breathing exercises are a cornerstone of the intervention, emphasized by their therapeutic potential^39,40^ and versatility across diverse clinical populations. Breathing involves the coordination of several systems in the body, including the respiratory, cardiovascular, and nervous systems. Several factors can impact breathing patterns, including age, gender, lung capacity, and overall health.^41,42^ By learning to be aware of our breathing, we can learn not to cling to thoughts and emotions, and this may lead to greater well-being overall.^42,43^ Techniques such as diaphragmatic, slow, rhythmic and mindful breathing but also alternate nostril breathing, have health benefits and are linked to both physical and mental functions.^39,40^ Various scientific reviews and meta-analyses demonstrate that deep breathing exercises activate the parasympathetic nervous system, facilitate relaxation and reduce stress.^44^ Slow and rhythmic breathing has been suggested to have a positive impact on anxiety and depression.^39,41,43^ Core exercises include mindful breath awareness, diaphragmatic breathing, slow rhythmic breathing, alternate nostril breathing and step breathing, which all are recognized for their potential to enhance mindfulness, autonomic balance, emotional regulation and overall well-being.^34,39,40,43^ Each breathing exercise can be tailored to meet individual needs, making them accessible to patients with various health conditions and capabilities. By fostering an intentional and mindful approach, the exercises provide a practical and therapeutic gateway to the psychophysiological benefits.^44^

#### Physical Exercises (Asana)

In yoga, Asanas are described as physical positions and movements to increase self-awareness and prepare the mind to expand.^1^ Physical exercises in this framework are to be considered as mindfulness-based physical activity with coordinated breathing. These exercises have the potential to enhance flexibility by promoting safe and gentle movements. The aim is to support joint and muscle mobility and contribute to functional strength and postural stability through low- to moderate-intensity activity that aligns with individual capabilities. Furthermore, the framework emphasizes the importance of balance, intentional movements that enhance physical stability, coordination, bodily control and includes a mixed balance of lying, sitting, and standing positions. To improve or maintain physical health,^45^ physical exercise can be accomplished with a structured, purposeful, and repetitive subset of physical activity, which can reduce the risk of developing non-communicable diseases (NCDs), such as heart diseases, diabetes, and cancer.^46^ Physical activity has also been shown to reduce depression and anxiety symptoms while increasing overall well-being.^46,47^ Furthermore, increasing duration of physical activity of any intensity, is associated with a reduced risk of premature mortality.^48,49^

#### Meditation Exercises (Dhyana)

In yoga, Dhyana refers to meditation^1^ which can be described as a concept or an exercise. Meditation exercises in this framework are to be considered as a mindfulness-based mix of awareness activities, emphasizing mental and emotional balance in enhancing emotional regulation, cognitive skills, mental clarity, and resilience. Techniques, such as breath-regulation, focus, counting, sounds/mantra, movement and mudras, engage and challenge observational and attentional skills.

Improvements in stress, anxiety, depression, pain, and immune function may be accomplished after conducting meditation exercises.^50,51^ Meditation is commonly described as a “self-regulation practice” aimed at training attention and awareness to foster calm, clarity, concentration and mental well-being.^52^ Mindfulness is recognized both as a meditation technique and a state or skill, *“paying attention in a particular way: on purpose, in the present moment, non-judgmentally*”.^53^ This state represents a natural human capacity that can be cultivated through practice and applied in daily life.^54^

There are several distinct categories of meditation as awareness activities. These can be structured into three primary categories: Focused Attention (FA), Open Monitoring (OM), and Compassion (CO).^54-56^ As a part of the concept of yoga itself, meditation carries a profound complexity and is further presented in the fourth interconnected layer.

### Awareness Layer - Mindful-Awareness Cycle (MAC)

MAC consists of three interconnected components that operate in a dynamic process.

#### Mindful Observation (Pratyahara)

In yoga, Pratyahara indicates withdrawing attention from external stimuli to cultivate inward focus, reducing distractions and fostering deeper presence.^1^ In this framework, Pratyahara is defined as mindful observation, akin to open awareness or OM. It involves non-reactive observation of sensory experiences and thoughts.^55^ Mindful Observation enhances awareness of physical, mental, and emotional states, revealing habitual mental patterns and potentially improving coping skills and emotional regulation.^50^ Research shows that OM meditation reduces default mode network (DMN) activity, associated with self-referential thoughts and mind-wandering,^57^ which can include daydreaming, planning, or rumination.^58,59^ While mind-wandering may support creativity and goal-oriented thinking, researchers propose that it can also contribute to conditions such as depression, anxiety, and attention deficit hyperactivity disorder (ADHD).^60^ Meditation practice may strengthen attentional control and executive functions that help regulate these processes.^61,62^

#### Mindful Attention (Dharana)

In yoga, Dharana means *“binding the mind to one object.*^1^ In this framework Dharana is defined as mindful attention which can be described as sustained awareness, focus and concentration and can be described as focused attention or FA. According to Lutz et al this awareness activity involves directing attention to a specific object, such as localized sensations caused by respiration, and continually monitoring the quality of attention.^55^ Attention skills can help to reduce mind-wandering by providing a mental anchor. The purpose is to reduce unintentional mind-wandering. By becoming more aware of when the mind starts to wander, the person can consciously redirect attention back to the present moment, thereby reducing the frequency and duration of mind wandering episodes.

#### Mindful Meta-Awareness (Samadhi)

In yoga, Samadhi is described as a shift into a deeper state of consciousness characterized by sustained absorption in the object of meditation and a loss of ordinary self-referential processing.^1^ Samadhi is operationalized, in this framework, as Mindful Meta-Awareness which can be described as awareness of awareness. Meta-awareness is considered essential across many forms of mind-body training as it is thought to play a key role in processes central to therapeutic effects in mindfulness-based interventions. It can be described both as a capacity and as a quality. The result may be to experience thoughts as mental events, and not as the solid things that they seem to represent.^63^ The construct of “Mindful Meta-Awareness,” recently proposed by Dunne et al as non-propositional and sustained, supports continuous and unified awareness beyond mental constructs.^63^ This perspective aligns contemporary mindfulness more closely with yoga, where Samadhi reflects sustained, non-dualistic meta-awareness, bridging contemporary practices with their holistic roots.

### Dynamic Interplay in the MAC Layer

The MAC layer integrates top-down mindful attention, bottom-up mindful observation and their convergence through mindful meta-awareness, drawing from PYSD and contemporary attentional and interoceptive models.^20,63-66^ Emerging from FA and OM, mindfulness research has increasingly moved toward describing mindfulness as an integrated process, aligning with the holistic nature of yoga and mind-body traditions. This dynamic and cyclical interplay supports mind-body regulation through integration of sustained meta-awareness and promotes self-regulation, resilience and progressive awareness skills.^64,67^

## The Integrated Theoretical Model

The integrated MOSI theoretical model illustrates how the previously defined layers and components interact within a coherent conceptual architecture:

1. The Basic Layer, Self-Care Aspects, which defines the behavioral and contextual foundation supporting sustainable lifestyle integration, adherence, and long-term engagement.
2. The Intentional Layer, Mindset, which defines the intentional and attitudinal orientation underlying intervention processes and is operationalized through core mindfulness qualities supporting adaptive psychological regulation.
3. The Practice Layer, Exercises, which serves as the primary intervention component and facilitates engagement through structured breathing, physical, and meditation exercises, all unified by the regulatory processes represented in the Awareness Layer.
4. The Awareness Layer, represented by the Mindful Awareness Cycle (MAC), which defines a dynamic regulatory process integrating observation, attention, and meta-awareness to support self-regulation and awareness development.

**Figure 3.**
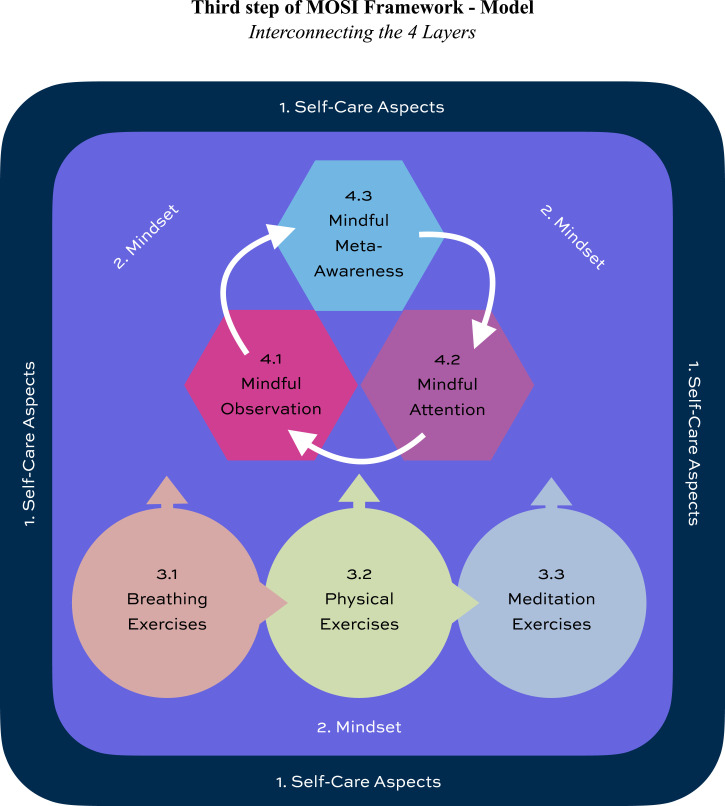
The integrated MOSI theoretical model

Figure 3 illustrates the integrated structure of the MOSI theoretical framework, showing how the four layers interconnect as a coherent and clinically operational architecture.

## Clinical Relevance and Future Perspectives

At this stage, the framework establishes: (I) a structured taxonomy of core intervention components; (II) standardized terminology for clinical and research applications; and (III) an integrated model that organizes relationships between components within a clinically applicable intervention structure (Figure 3).

By providing a shared conceptual structure and standardized terminology, the MOSI framework addresses key methodological barriers that have limited consistent intervention specification, scalability, interdisciplinary communication, and implementation across clinical contexts.

As a theoretical framework, MOSI provides a conceptual foundation for systematic intervention design, which may strengthen methodological clarity, reproducibility, and research capacity by aligning traditional yoga practices with clinical requirements. This alignment may also improve intervention fidelity, support scalable implementation, and strengthen the methodological rigor of yoga-based clinical research.

MOSI may also facilitate scalable training of healthcare professionals, support cumulative evidence development across diagnostic areas, and provide a stable intervention architecture while allowing context-specific adaptations.

The structured progression of the MOSI framework also resonates with pedagogical principles used in established mindfulness-based interventions, reflecting the growing convergence between yoga-based and mindfulness-based approaches in clinical research.

Together with reporting guidelines such as CLARIFY 2021, the MOSI framework may strengthen methodological consistency and support more precise specification and reporting of yoga-based interventions in healthcare research.

Future work should focus on empirical validation of the framework, development of intervention protocols and clinical guidelines informed by the MOSI structure, and evaluation of its utility in improving intervention reproducibility, reporting quality, and clinical implementation.

## Conclusion

The systematic integration of yoga into healthcare requires clear theoretical taxonomy, standardized terminology, and operational definitions to support consistent clinical implementation and scientific evaluation.

The MOSI theoretical framework addresses this need by providing a structured taxonomic derivation of traditional yoga theory into clinically operational theoretical architecture. By defining intervention components, operational terminology, and an integrated theoretical model, MOSI establishes a conceptual foundation for standardized and reproducible intervention design and systematic implementation in clinical contexts.

Complementing existing reporting guidelines such as CLARIFY 2021, the MOSI framework may support methodological consistency and provide a structural foundation for the development, empirical validation and implementation of yoga-based interventions within modern healthcare.
